# Inhibitor of Differentiation-2 Protein Ameliorates DSS-Induced Ulcerative Colitis by Inhibiting NF-κB Activation in Neutrophils

**DOI:** 10.3389/fimmu.2021.760999

**Published:** 2021-11-04

**Authors:** Jie Ren, Dong Yan, Yichun Wang, Jiaojiao Zhang, Min Li, Wancheng Xiong, Xueqian Jing, Puze Li, Weidong Zhao, Xiwen Xiong, Minna Wu, Genshen Zhong

**Affiliations:** ^1^ School of Basic Medicine, Xinxiang Medical University, Xinxiang, China; ^2^ Henan Key Laboratory of Immunology and Targeted Therapy, Henan Collaborative Innovation Center of Molecular Diagnosis and Laboratory Medicine, School of Laboratory Medicine, Xinxiang Medical University, Xinxiang, China; ^3^ The First Affiliated Hospital of Xinxiang Medical University, Xinxiang, China; ^4^ School of Forensic Medicine, Xinxiang Medical University, Xinxiang, China

**Keywords:** Inhibitor of differentiation-2 (ID2), ulcerative colitis (UC), intestinal barrier, neutrophils, nuclear factor kappa B (NF-κB), recombinant protein

## Abstract

The loss of inhibitor of differentiation-2 (ID2) could lead to the development of colitis in mice, supplementation with exogenous ID2 protein might be a potential strategy to ameliorate colitis. In this study, the effects of ID2 protein supplementation on Dextran sodium sulfate (DSS)-induced colitis were investigated. Firstly, we confirmed that the expression of ID2 was reduced in the colon tissues of DSS-induced colitis mice and patients with ulcerative colitis (UC). Then, we constructed a recombinant plasmid containing the human *Id2* gene and expressed it in *Escherichia coli* (*E. coli*) successfully. After purification and identification, purified hID2 could ameliorate DSS-induced colitis efficiently in mice by improving disease symptoms, decreasing the levels of proinflammatory cytokines in colon tissues, maintaining the integrity of intestinal barrier and reducing the infiltration of neutrophils and macrophages in the colon. Further study showed that hID2 could be endocytosed efficiently by neutrophils and macrophages, and hID2 lost its protection function against colitis when neutrophils were depleted with an anti-Gr-1 antibody. hID2 decreased the mRNA levels of IL-6, IL-1β and TNF-α in lipopolysaccharides (LPS)-stimulated neutrophils and efficiently inhibited the activation of NF-κB signalling pathway in neutrophils. Interestingly, hID2 showed a synergistic role in inhibition of NF-κB activation with pyrrolidine dithiocarbamic acid (PDTC), an inhibitor of NF-κB activation. Therefore, this study demonstrated the potential use of hID2 to treat UC, and hID2 protein might be a promising anti-inflammatory agent that targets the NF-κB signalling pathway in neutrophils.

## Highlights

1. This study demonstrated the expression of ID2 protein was decreased in DSS-induced colitis mice and UC patients;
2. Exogenous supplementation of recombinant human ID2 protein (hID2) could ameliorated DSS-induced colitis in mice, the protection of which was depended on neutrophils;
3. Recombinant human ID2 protein (hID2) suppressed the secretion of pro-inflammatory cytokines through inhibiting the activation of IκB/NF-κB pathway in LPS-stimulated neutrophils.

## Introduction

Ulcerative colitis (UC), an inflammatory bowel disease (IBD), is an idiopathic, chronic inflammatory disorder of the mucosa and submucosa of the rectum and colon (1). Bloody diarrhoea and abdominal pain are the main characteristic symptoms of UC (1), Until now the precise cause of UC is still far from clear. Genetic susceptibility, dysfunction of the gut microbiota, dysregulation of the immune response, disruption of colon barrier function and leucocyte recruitment are all the possible factors for resulting the occurrence and development of UC disease (2).

Recently, studies have indicated that abnormal responses of the innate and adaptive immune systems, and dysregulation of the gut microbiota might lead to an aberrant intestinal inflammatory response in patients with IBD. In UC patients, the Th17/Treg balance is important for homeostasis of the cellular niche in the colon, and other subsets of T cells, such as Th1 and Th2 cells, also play roles in the development and maintenance of inflammation (3, 4). The innate immune system is also important for maintaining intestinal homeostasis. Mononuclear phagocytes, particularly macrophages, are essential for maintaining gut homeostasis and protecting against certain pathogens in the steady phase. However, the number of macrophages in the intestinal lamina propria (LP) increases in the active phase of UC and causes immunopathology (5, 6). Activated macrophages produce excessive proinflammatory factors such as IL-1β, IL-6 and TNF-α, which not only contribute to inflammatory responses by stimulating neutrophil migration and activating other lymphocytes but also promote intestinal epithelial tissue damage, which further increases the invasion of pathogenic intestinal bacteria (7–9). There is increased infiltration of neutrophils and macrophages, increased intestinal permeability, and the loss of expression of tight junction proteins such as ZO-1 and Claudin-1 in colon inflammatory sites in UC patients and colitis mice (10–12). In addition, the degree to which neutrophils infiltrate the epithelium and lamina propria (LP) reflects the severity of UC (13), and delayed neutrophil apoptosis was observed in UC, which was attributed to the release of antiapoptotic cytokines such as granulocyte-macrophage colony-stimulating factor (GM-CSF) (14, 15). These results suggest that the uncontrolled accumulation of neutrophils and macrophages and their persistent presence in LP may exacerbate the development of intestinal inflammation.

The transcription factor NF-κB is a classic mediator of inflammation that mediates the interaction of cells, cell survival and differentiation, and the expression of cytokines, chemokines and coagulation factors (16). Studies have shown that NF-κB overactivation plays a key role in the pathogenesis and progression of UC in mice or humans and is associated with abnormal levels of intestinal inflammation (17–19). In UC patients, the activation of NF-κB in LP macrophages led to the secretion of proinflammatory cytokines and caused the progression of inflammation (20). Moreover, inhibition of the NF-κB pathway can induce neutrophil apoptosis, thereby alleviating inflammatory injury (21). Therefore, promoting timely clearance or decreasing the infiltration of macrophages and neutrophils in the LP, inhibiting overactivation of the NF-κB pathway in macrophages or neutrophils may be an effective strategy for treating UC.

Treatment strategies for UC currently include specific and nonspecific drugs (corticosteroids, 5-aminosalicylates, folic acid inhibitors, anti-TNF-α or anti-integrin antibodies and antibiotics), faecal microbiota transplantation (FMT) and surgery (22). However, due to the limitations of current treatment methods and the low response rates in certain UC patients, more efficient and safe methods of treating UC are urgently needed.

Inhibitor of differentiation (ID) belongs to the helix-loop-helix transcription factor family, which inhibits the binding of nuclear transcription factors to DNA. ID proteins are widely found in mammals and have four family members (ID1-ID4) (23). Inhibitor of differentiation-2 (ID2) is a helix-loop-helix (HLH) transcription factor that not only plays an important role in adaptive immune cells such as T cell subsets and B cells, but is also involved in the differentiation of innate immune cells, including neutrophils, dendritic cells (DCs), natural killer (NK) cells, and innate lymphoid cells (ILCs) (23). Loss of ID2 expression increases adipose regulatory T cells (aTregs) cell death due to increased expression of Fas. At the same time, ID2-mediated aTreg loss leads to increased systemic inflammation (24). The reduced expression of ID2 and ID3 in Treg cells leads to impaired maintenance and localization of Treg cell population (25). ID2 also plays an important role in the development of gastrointestinal epithelial region-specificity and determines intestinal characteristics by inhibiting the foregut transcription factor Irx5 (26). Inhibition of ID1 and ID2 gene expression resulted in apoptosis of rat intestinal epithelial cells, while overexpression of ID2 attenuated TGF-β-induced apoptosis (27). In addition, ID2 is essential for the intestinal mucosal barrier, and mice with *Id2* and *Id3* depletion develop colitis (28, 29). Therefore, it seems that ID2 expression is highly correlated with the prognosis of colitis, the role of exogenous ID2 protein supplementation in the treatment of UC has not been investigated, and whether this protein affects the function of neutrophils and macrophages is still unknown. In this study, we showed that exogenous supplementation with ID2 protein could efficiently protect mice against DSS-induced colitis by inhibiting NF-κB activation in neutrophils.

## Materials and Methods

### Construction of the pET-30a (+)/*hId2* Plasmid and Preparation of the hID2 Protein

*E. coli* DH5α and pET-30a (+) plasmids were purchased from Invitrogen. According to the reported *Id2* gene sequence and restriction site analysis, a pair of primers was designed by Invitrogen. The primer sequences of *Id2* were as follows: forward: 5’-GGGAATTC **CATATG** AAAGCCTTCAGTCCCGT-3’; reverse: 5’-CCG **CTCGAG** GCCACACAGTGCTTTGCTGT-3’; the underlined regions are the restriction sites of Nde I and Xho I, respectively. The human *Id2* gene sequence was used as a template for PCR amplification, and the sequences of human *Id2* genes are provided in **Figure S1**. The reaction conditions were as follows: predenaturation at 94°C for 5 minutes, denaturation at 94°C for 2 minutes, annealing at 60°C for 30 seconds and elongation at 72°C for 40 seconds, for a total of 30 cycles. Then, extension was performed at 72°C for 10 minutes. Agarose gel electrophoresis was carried out for the obtained PCR products, and the DNA fragment glass milk recovery kit was purchased from BioDev-Tech. Co., Ltd. and used for gel recovery and purification. Both the purified PCR products and pET-30a (+) plasmid were used for Nde I/Xho I double digestion. Then, T_4_ DNA ligase was used to connect the *Id2* fragment and plasmid and convert the product to *E. coli* DH5α to construct the pET-30a (+)/*hId2* recombinant plasmid.

The constructed pET-30a (+)/*hId2* recombinant plasmids and no-load plasmids were transformed into *E. coli* BL21(DE3), and a single colony was selected from the transformation plate and inoculated into Luria-Bertani medium containing 30 μg/ml kanamycin, which was shaken at 37°C. Then, IPTG (isopropyl-β-D-thiogalactoside) was added to the culture and incubated for another 3 hours to induce high expression of recombinant human ID2 protein. The preparation of recombinant protein was described in our previous study (30). The bacteria were collected by centrifugation and broken by ultrasound. The supernatant was then discarded, and the precipitate was resuspended in the binding buffer containing 2 M urea, the process was repeated three times. Finally, a binding buffer containing 6 M urea was used to resuspend the precipitate, which was placed on ice to dissolve for one hour. The supernatant was collected after centrifugation at 12,000×g for 30 minutes. The supernatant was filtered through a 0.45 µm filter membrane and then purified on a Ni^+^ affinity agarose gel. The specific steps are as follows: sterile deionized water, charge buffer containing 50 mM NiSO_4_ and binding buffer containing 6 M urea were added to fill the balanced column. The collected supernatant was then loaded and allowed to permeate freely, and the filtered supernatant was collected. Next, the column was washed with binding buffer containing 6 M urea and washing buffer containing 60 mM imidazole, and the washing liquid was collected. Finally, the binding protein was eluted with solution containing 250 mM or 1 M imidazole and collected separately. The eluent collected during the above steps was analysed by SDS-PAGE. Next, different concentrations of urea solutions were used for step-by-step dialysis to renature the purified protein. Finally, the dialyzed protein was centrifuged at 10,000×g for 30 minutes, and the supernatant was collected. The supernatant was added to an ultrafiltration centrifuge tube with a 5,000 Dalton cut-off and centrifuged at 5,000×g for 15 minutes to concentrate the protein. To remove possible residual endotoxin contamination, Triton X-114 was used to reduce the residual endotoxin by a phase separation technique as previously described (31).

### Protein Concentration, Identification, and FITC Labelling

The recombinant human ID2 protein concentration was determined according to the instructions of the BCA protein assay kit. SDS–PAGE and Western blot analysis were then performed, and protein identification was performed using the gel separated hID2 by Shanghai Applied Protein Technology Co., Ltd. to identify the recombinant human ID2 protein. In brief, the cutted gel containing the targeting band was digested in trypsin solution at 37°C for 12h, and the tryptic peptides were extracted with 60% acetonitrile and 0.1% trifluoroacetic acid. The dry peptide samples were reconstituted in standard diluent, spotted on Opti-TOF stainless steel plate, covered with matrix and air dried. Each fraction was injected into a Q-Exactive mass spectrometer (Thermo Scientific) for LC-MS/MS analysis. Peptide mass fingerprinting and MS/MS queries were performed using the MASCOT engine (Matrix Science, London, UK; version 2.4) (32, 33). The method for labelling hID2 with FITC was previously described (30). In brief, the recombinant human ID2 protein and FITC were shaken at 4°C in carbonate buffer solution [100 mmol/L NaHCO_3_, 10 mmol/L Na_2_CO_3_ (pH 9.0)] for 12 hours, and then the FITC-labelled hID2 protein was isolated in a Millipore hyperfiltration tube with a cut-off of 5,000 Daltons.

### Animals

Six-week-old male C57BL/6J mice were purchased from Zhejiang Vital River Laboratory Animal Technology Co., Ltd. (Zhejiang, China). The mice were housed for 1 week before the experiment to adapt to the environment (weight: 20–22 g). The mice were housed in individually ventilated caging (IVC) systems under a 12-h light/dark cycle at an environmental temperature of 23°C ± 2°C and humidity of 55% ± 5% and allowed free access to sterilized standard rodent chow and sterilized water. The animal experiments were conducted in accordance with the principles provided by the National Institutes of Health Guide for the Care and Use of Laboratory Animals and approved by the Ethics Committee of Xinxiang Medical University.

### Experimental Ulcerative Colitis Model and Treatment

To investigate the role of hID2 in the development and progression of ulcerative colitis in mice, the mice were randomly divided into four groups (n=6 for each group): a blank control group (CON group), a recombinant human ID2 protein-treated control group (hID2 group), a DSS-induced UC model group (DSS group), and a hID2-treated UC group (DSS+hID2 group). Dextran sulfate sodium (DSS, molecular weight of 36–50 kDa) was purchased from MP Biochemicals (Santa Ana, CA, USA) and dissolved in drinking water, and acute colitis was induced with 2.5% DSS in drinking water for 7 days. Recombinant human ID2 protein (5 mg/kg) was given intraperitoneally on the third day and fifth day after the DSS-induced colitis. On the 8^th^ day, the mice were anaesthetized and sacrificed.

### Calculation of Disease Activity Index (DAI)

As previously described, the severity of colitis was assessed by the disease activity index (DAI) score, which was derived from the average sum of the weight loss score, rectal bleeding score, and diarrhoea score (34, 35). The body weights of the mice were recorded every day during the experiment. Rectal bleeding was scored according to the instructions of the faecal occult blood kit, which was obtained from Baso Biotechnology Co., Ltd. (Guangdong, China).

### Histopathological Assessment, Immunohistochemistry and Immunofluorescence Analysis

Distal colon tissues were fixed with 4% paraformaldehyde before being embedded in paraffin. To assess inflammation, colon tissue cross sections were stained with haematoxylin and eosin (H&E) (Beyotime, Shanghai, China). The pathological score was assessed as previously described (36, 37). Briefly, the total score was calculated from the inflammatory cell infiltration score (0–4), the mucosal thickening score (0–4), the goblet cell depletion score (0–4), the structure destruction score (0 or 3–4) and the crypt loss score (0 or 3–4). Colon sections were embedded in paraffin, and neutrophils were fixed in 4% paraformaldehyde and blocked with BSA. Neutrophils were permeabilized with 1% Triton X-100 and incubated with PE-labelled anti-mouse Ly6G antibodies (BD Biosciences, clone: 1A8), PE-labelled anti-mouse F4/80 antibodies (BD Biosciences, clone: BM8) and phosphorylated p65 (p-p65) antibodies (Cell Signaling Technology, Inc, clone: 14D4). Anti-rabbit secondary antibodies were added, and the cells were counterstained with 4’,6-diamidino-2-phenylindole (DAPI). At the same time, immunohistochemistry was performed according to the streptavidin-peroxidase (SP) method according to the instructions, and the colon sections were incubated with anti-mouse/human ID2 antibodies (Abcam, ab85990). All images were taken by a fluorescence microscope (Leica, Germany) under the same exposure and intensity settings.

### Reverse Transcription Quantitative Polymerase Chain Reaction (RT-qPCR) Analysis

Total RNA was extracted from colonic tissues using TRIzol reagent (Tiangen, Beijing, China). Then, the isolated total RNA was reverse transcribed into cDNA using PrimeScript RT master mix (Takara, Dalian, China). Quantitative PCR analysis was performed using SYBR Green PCR Core Reagent (Takara, Dalian, China) in an ABI StepOnePlus Real-Time PCR system. The primers were synthesized by Takara Biotechnology Co., Ltd. (Takara, Dalian, China). The housekeeping gene glyceraldehyde-3-phosphate dehydrogenase (GAPDH) was used as a reference gene. The gene (mRNA) expression levels were normalized to GAPDH in the same sample, and the relative expression levels were calculated using the ^ΔΔ^Ct method (38). The primer sequences used for RT–qPCR analysis is shown in **Table S1**.

### Western Blot Analysis

Colon tissues or neutrophils were homogenized in RIPA buffer with protease inhibitor buffer (1:100) and phosphatase inhibitors (1:50). The protein concentration was determined with a BCA protein assay kit (Thermo Fisher Scientific, Waltham, MA, USA). Equal amounts of protein were separated by SDS–PAGE and transferred onto nitrocellulose membranes. After being blocked with 5% nonfat milk in TBST, the membranes were incubated at 4°C overnight with rabbit polyclonal antibodies against α-Actinin (1:5,000), ZO-1 (1:1,000), Claudin-1 (1:800) and IκBα (1:1,000) (Proteintech Group), p-IκBα (1:1,000), NF-κB (p65) (1:1,000), p-NF-κB (p65) (1:1,000) (Cell Signaling Technology), and β-actin (1:5,000) (Santa Cruz Biotechnology). Then, the nitrocellulose membranes were incubated at room temperature for 1.5 hours with goat anti-rabbit horseradish peroxidase (HRP)-conjugated secondary antibodies (1:5,000), which were obtained from ZSGB-BIO, Inc. (Beijing, China). Finally, ImageJ software was used for optical density analysis. And the uncropped and representative images were shown in **Supplementary Materials** (**Figures S2, S3**).

### Flow Cytometry and Absolute Cell Counts

Spleen cells from mice in each group were separated by grinding on filters. Peripheral blood was collected from the mice into EDTA anticoagulant tubes. Red blood cells were lysed using red blood cell lysis buffer (C3702; Beyotime, China). The whole colon was digested by collagenase VIII and DNase I for 1.5 hours to obtain an LP single-cell suspension. The absolute cell count method was performed according to the instructions of the 123count™ eBeads (Thermo Fisher, Cat#:01-1234-42). In brief, the cells and eBeads were mixed, and the following equation was used to calculate absolute counts: Absolute Count (cells/μl) = (Cell Count/eBeads Count) × eBeads Concentration. The eBeads were visualized with fluorescent parameters such as FITC and PE. For cell surface marker staining, cells were first incubated with serum for Fc receptor blocking and then incubated with fluorescence-conjugated antibodies, such as FITC-labelled anti-CD45 (Thermo Fisher Scientific, clone: 30-F11), PerCP/Cy5.5-labelled anti-CD11b (Thermo Fisher Scientific, clone: M1/70), PE-labelled anti-Ly6G (BD Biosciences, clone: 1A8), APC-labelled anti-F4/80 (BD Biosciences, clone: BM8), PerCP/Cy5.5-labelled anti-CD3 (BD Biosciences, clone: 17A2), and FITC-labelled anti-CD4 (BioLegend, clone: GK1.5). For intracellular staining, cells were fixed and permeabilized with an eBioscience Intracellular Fixation & Permeabilization Buffer Set (Thermo Fisher Scientific, Waltham, MA, USA), and then PE-labelled anti-Foxp3 (Thermo Fisher Scientific, clone: FJK-16S) and APC-labelled anti-RORγt (Thermo Fisher Scientific, clone: AFKJS-9) were used. After being washed with PBS, the cells were analysed on a BD FACSCanto flow cytometer system, and the obtained data were analysed with FlowJo 10.0 software.

### Total Faecal DNA Isolation and 16S rDNA Gene High-Throughput Sequencing

Total DNA was isolated from fresh stool samples according to the procedures of the DNA Stool Kit (Biomiga, Shanghai, China). The purity and concentration of DNA were determined by a NanoDrop 2000 ultraviolet spectrophotometer. The V3-V4 regions were amplified by the 16S rDNA gene universal primers 338F (5’-ACTCCTACGGGAGGCAGC-3’) and 806R (5’-GGACTACHVGGGTWTCTAAT-3’) (39). Then, the PCR products were purified, and the concentration was adjusted. An Illumina MiSeq PE300 system (MajorBio Co., Ltd, Shanghai, China) was used for sequencing. The accession number is SUB10228064.

### Isolation of Bone Marrow Cells and Cell Culture

The mice were sacrificed and then exposed to 75% alcohol for 5 minutes. The bones of the four limbs were removed under sterile conditions, the muscle was removed, and the bone marrow cells were flushed out with PBS and filtered through a 200-mesh filter. Furthermore, Percoll cell separation solution was used to isolate neutrophils from the bone marrow. A 100% Percoll separation solution was prepared with Percoll original solution and 10× PBS (9:1). Then, 3 ml of the Percoll separation solution at 78% and 65% concentrations was placed into a 15 ml centrifuge tube. Finally, 3 ml of the cell suspension was added slowly and centrifuged at 800×g for 35 mins. The white layer was the neutrophilic layer, and the purity of the separated neutrophils was determined by flow cytometry. The red blood cells were lysed, washed twice with PBS, and counted by an abalone counting plate. Neutrophils were cultured in RPMI 1640 and matured with GM-CSF. Caco-2 cells were obtained from the American Type Culture Collection (ATCC) and cultured in DMEM. All media contained 10% foetal bovine serum and 1% penicillin and streptomycin.

For the internalization of FITC-labelled hID2, the isolated bone marrow or spleen cells were counted and resuspended in PBS, and then different concentrations of FITC-hID2 were added and incubated at 37°C for 1 h. Finally, 0.4% trypan blue was added to quench the extracellular fluorescence, and the cells were washed with PBS three times. In addition, antibodies were used for staining and flow detection.

For the treatment of Caco-2 cells with DSS (40), Caco-2 cells were transferred into 96 wells and treated with different concentrations of DSS (1% to 5%). After 24 hours, the CCK-8 assay was used to determine the proliferation of Caco-2 cells. In addition, to investigate the effect of hID2 on Caco-2 cells, Caco-2 cells were treated with different concentrations of hID2 for 24 hours, and then cell proliferation was measured directly or 3% DSS was added and incubated for 24 hours to determine changes in cell proliferation.

### Intragastric Gavage of FITC-Dextran and Salmonella Invasion Assays *In Vivo*

The FITC-labelled dextran method was used to assess the intestinal barrier permeability in mice and was modified from a previous description (41). Briefly, the mice were gavaged with 500 mg/kg FITC-dextran (molecular weight 3,000–5,000; Sigma–Aldrich). After 4 h, the serum was collected and protected from the light. The detection wavelength was 490 nm, and the emission wavelength was 530 nm. The serum concentration of FITC-dextran was calculated according to the standard curve.

To test intestinal barrier function, the experimental mice were orally administered 2×10^9^ colony-forming units (CFUs) of Salmonella strains (NCTC12023) containing an ampicillin-resistance plasmid (pFPV25.1) as described previously (42). At 4 hours after administration, the entire colon was collected, washed with cold PBS buffer and weighed. Then, the colon was homogenized with 1 ml of cold PBS, and the colonic suspensions were successively diluted ten times. Then, the suspensions were coated on a solid Luria-Bertani plate containing ampicillin and incubated overnight at 37°C, and the colonies were counted.

### Statistical Analysis

All data are expressed as the mean ± standard error of the mean (SEM). GraphPad Prism 5.00 software was used for data analysis. Statistical results were evaluated using unpaired Student’s t-test or one-way analysis of variance (ANOVA), and *p* < 0.05 was considered statistically significant.

The 16S rDNA sequencing data were analysed on the free online Majorbio I-Sanger Cloud Platform (www.i-sanger.com). The operational taxonomic units (OTUs) were determined using a cut-off of 97% similarity by UPARSE (43) (version 7.1 http://drive5.com/uparse/), and chimeric sequences were identified and removed using UCHIME. Alpha diversity was measured according to community diversity and richness based on the observed OTU number with the PD and Chao index, and Student’s t-test was used to analyse the differences between the index groups. ANOSIM (analysis of similarities) uses the distance algorithm (Bray-Curtis) to calculate the distance between two samples. Beta diversity was determined by principal component analysis (PCA) based on the distance matrix.

## Results

### Decreased Expression of ID2 in UC Patients and Mice With DSS-Induced Colitis

**Figure 1:**
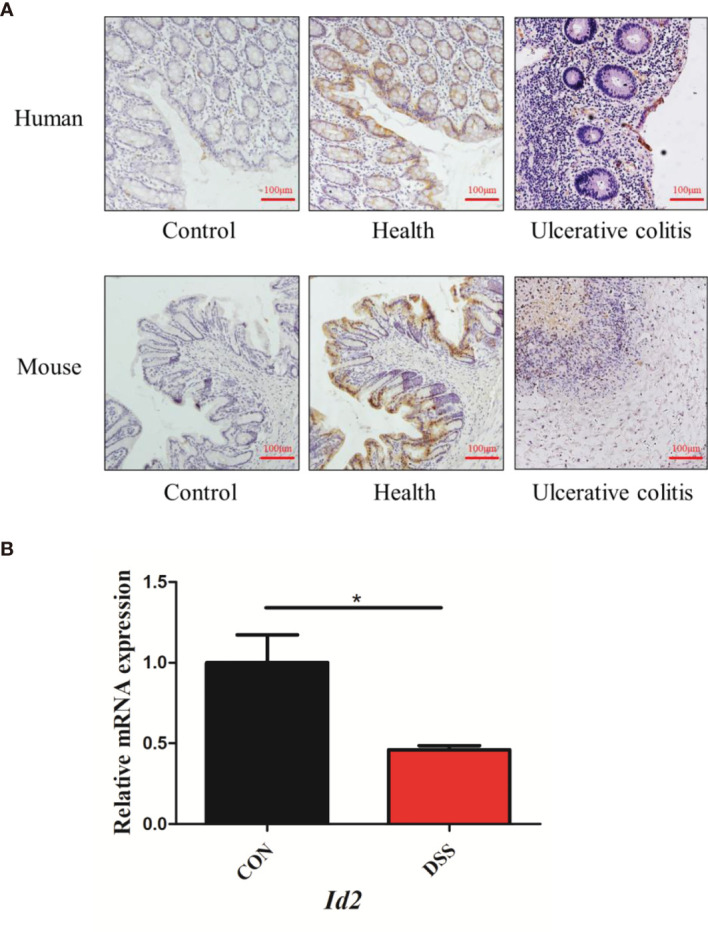
The expression of ID2 was decreased in the inflamed colons of UC patients and colitis mice. **(A)** Immunohistochemical staining of ID2 in colonic tissues (upper: colonic tissues of normal or UC patients; lower: colonic tissues of normal or UC mice) (200×). **(B)** The mRNA expression of Id2 in the colonic tissues of UC mice. The experiment was repeated three times independently. The data are presented as the mean ± SEM (n=3). ^*^ *p* < 0.05 *vs.* the control group.

ID2 expression is decreased in colitis. It was reported that mice lacking *Id2* and *Id3* could develop colitis (28). However, the expression of ID2 during colitis is not clear. To confirm the changes in ID2 in mice with UC, immunohistochemistry (IHC) and RT–qPCR were used. We found that ID2 was mainly expressed in the mucosal epithelium in healthy colon tissue and was decreased in the inflammatory site of the colon in UC patients or mice with DSS-induced colitis (**Figure 1A**). RT–qPCR analysis of mRNA isolated from the colons of DSS-induced mice also showed the decreased expression of *Id2* (**Figure 1B**). Our results further confirmed that ID2 might play a key role in the development progression of UC.

### Preparation of Recombinant hID2

**Figure 2:**
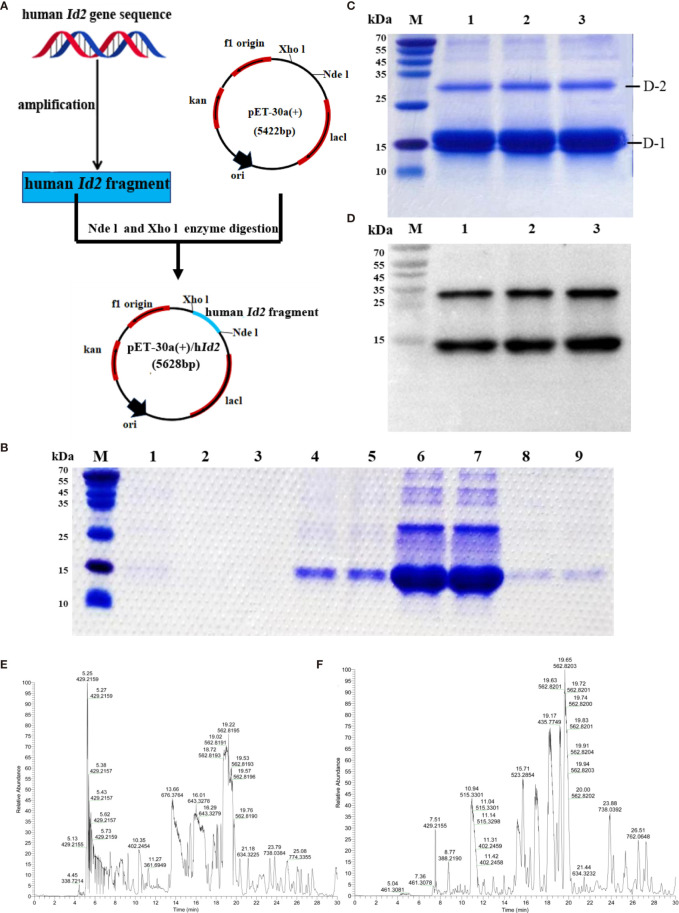
Preparation and identification of recombinant human ID2 protein (hID2). **(A)** Construction of the recombinant plasmid pET-30a (+)/*hId2*. **(B)** Coomassie Bright Blue analysis of purified hID2 protein (M: maker; Channel 1: free filter collection; Channel 2 and 3: Binding Buffer (without imidazole) wash collection; Channel 4 and 5: Washing Buffer (with 60 mM imidazole) wash collection; Channel 6 and 7: Low concentration Elution Buffer (with 250 mM imidazole) wash collection; Channel 8 and 9: High concentration Elution Buffer (with 1 M imidazole) wash collection). **(C)** Coomassie Bright Blue analysis of concentrated recombinant human ID2 protein after renaturation (channels 1–3 represent identical samples). **(D)** Western blot analysis of recombinant human ID2 protein using the anti-ID2 mAb (channels 1–3 represent identical samples). Mass spectrometric results of bands **(E)** D-1 and **(F)** D-2. The gel was cut and collected for LC-QE/MS analysis.

Human ID2 protein was produced successfully. To investigate the function of the ID2 protein in the development of colitis, we generated the recombinant ID2 protein. We constructed the recombinant plasmid pET-30a (+)/*hId2*, and the detailed flow chart is shown in **Figure 2A**. The recombinant plasmid was transferred into *Escherichia coli* BL21 (DE3), and IPTG induced a large amount of hID2 expression. The recombinant protein was purified by Ni^+^ affinity purification using the 6*His-tag (**Figure 2B**). SDS–PAGE and Western blot analysis using anti-ID2 antibody (**Figures 2C, D**) confirmed that the recombinant protein had good purity, and the relative molecular weight was approximately 15 kDa. Moreover, the gel was cut and sent for mass spectrometry analysis, and the results confirmed that the obtained protein was recombinant human ID2 protein and that the hID2 protein existed as a monomer and dimer format (**Figures 2E, F**).

### Recombinant hID2 Alleviated Ulcerative Colitis Induced by DSS in Mice

**Figure 3:**
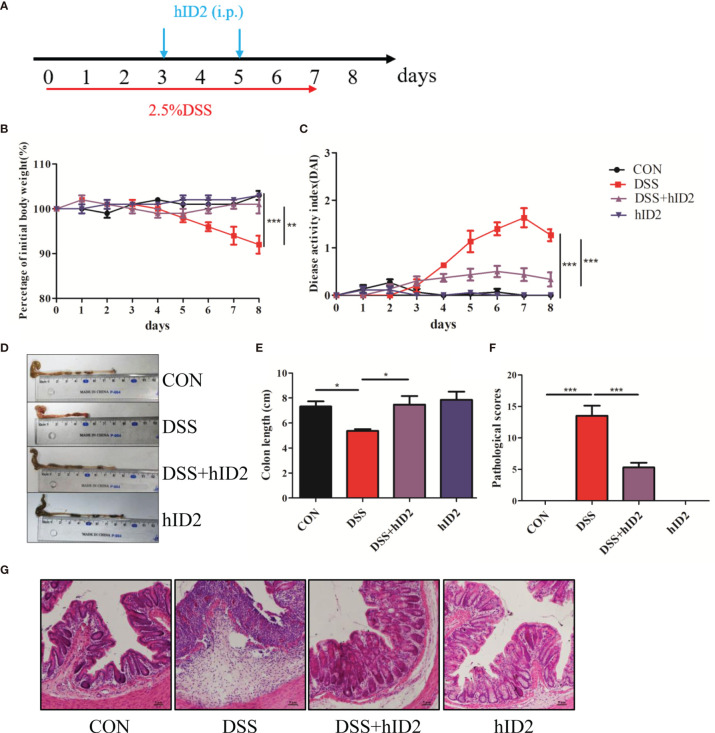
Recombinant human ID2 protein treatment ameliorated DSS-induced colitis in mice. **(A)** The experimental procedure; the arrow indicates the *i.p.* injection time point of hID2 during the DSS-induced colitis experiment. **(B)** Daily body weight changes. **(C)** Disease activity index (DAI) during the experiment. **(D)** Representative image of the colon and **(E)** colon length. **(F)** Pathological scores of colonic tissue sections and **(G)** H&E staining (200×). The experiment was repeated three times independently. The data are presented as the mean ± SEM (n=5). ^*^ *p* < 0.05, ^**^ *p* < 0.01, ^***^ *p* < 0.001.

To evaluate the role of ID2 in DSS-induced ulcerative colitis, 2.5% DSS was added to drinking water for 7 days to induce a mouse model of UC. During the experiment, exogenous hID2 protein was administered twice by intraperitoneal injection (**Figure 3A**). During the course of the experiment, DSS treatment resulted in weight loss, diarrhoea and haematochezia. DSS group mice had lower body weights and higher DAI scores than CON group mice, while hID2 administration decreased weight loss and the DAI scores in the DSS+hID2 group (**Figures 3B, C**). The colon length was longer in the DSS+hID2 group than that in the DSS group (**Figures 3D, E**). Furthermore, the results of H&E staining showed that hID2 could decrease the pathological scores of colon tissue (**Figures 3F, G**). Thus, hID2 treatment significantly ameliorated the disease symptoms of UC in mice induced by DSS.

### Recombinant hID2 Protected the Integrity of the Colonic Mucosal Barrier

**Figure 4:**
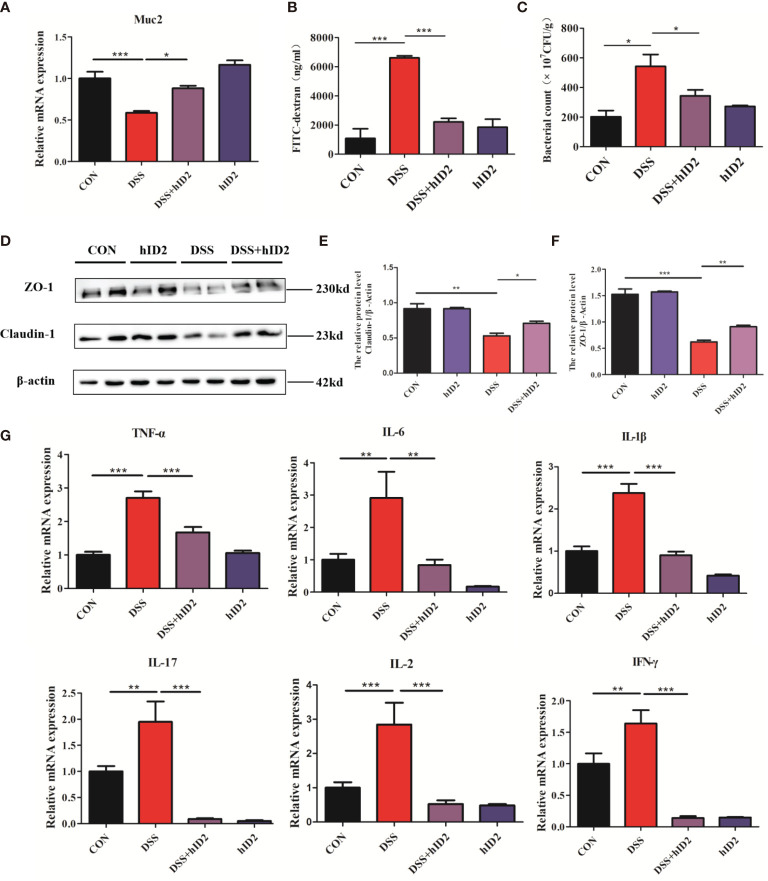
The effects of hID2 protein on intestinal barrier integrity and the reduction in inflammatory cytokines in colonic tissues. **(A)** The mRNA levels of *Muc2* in colonic tissues. **(B)** The concentrations of FITC-dextran in mouse serum. **(C)** Salmonella invasion assays in the colonic tissues of mice. **(D)** The protein expression of ZO-1 and Claudin-1 in colonic tissues. **(E)** Relative protein levels of Claudin-1. **(F)** Relative protein levels of ZO-1. **(G)** The mRNA levels of TNF-α, IL-6, IL-1β, IL-17, IL-2, and IFN-γ in colonic tissues. The data are presented as the mean ± SEM (n=5). The experiment was repeated three times independently. ^*^ *p* < 0.05, ^**^ *p* < 0.05, ^***^ *p* < 0.001.

hID2 showed robust protective effects against DSS-induced colitis, as reflected by improvements in the mucosal barrier. The mucosal barrier plays an important role in protecting against DSS-induced colitis in mice. To assess the effects of hID2 on the colonic mucosal barrier, the level of *Muc2* mRNA was measured, and the results indicated that the expression of *Muc2* was decreased in the colon in the DSS group compared with the CON group (**Figure 4A**, *p*<0.05). Moreover, the protein expression of Claudin-1 and ZO-1 was also decreased significantly in the DSS group (**Figures 4D–F**). However, hID2 administration increased the level of *Muc2* mRNA and the expression of Claudin-1 and ZO-1 (**Figures 4A, D**). Salmonella invasion experiments showed that the number of Salmonella in the colon in the DSS group was higher than that in the DSS+hID2 group (**Figure 4C**). In addition, the serum level of FITC-dextran in the DSS+ hID2 group was much lower than that in the DSS group and close to that in the CON group (**Figure 4B**).

### Recombinant hID2 Inhibited the Secretion of Proinflammatory Cytokines in the Colon

The production of proinflammatory mediators, including IL-1β, IL-6, TNF-α, IL-2, IL-17, and IFN-γ, plays a critical role in DSS-induced colitis in mice. To investigate the effect of hID2 on the mRNA levels of inflammation-associated cytokines, RT–qPCR analysis was performed. As shown in **Figure 4G**, DSS group mice had higher mRNA levels of proinflammatory cytokines compared with the DSS+hID2 group mice.

### Recombinant hID2 Reduced the Number of Inflammatory Cells in the Inflamed Colon

**Figure 5:**
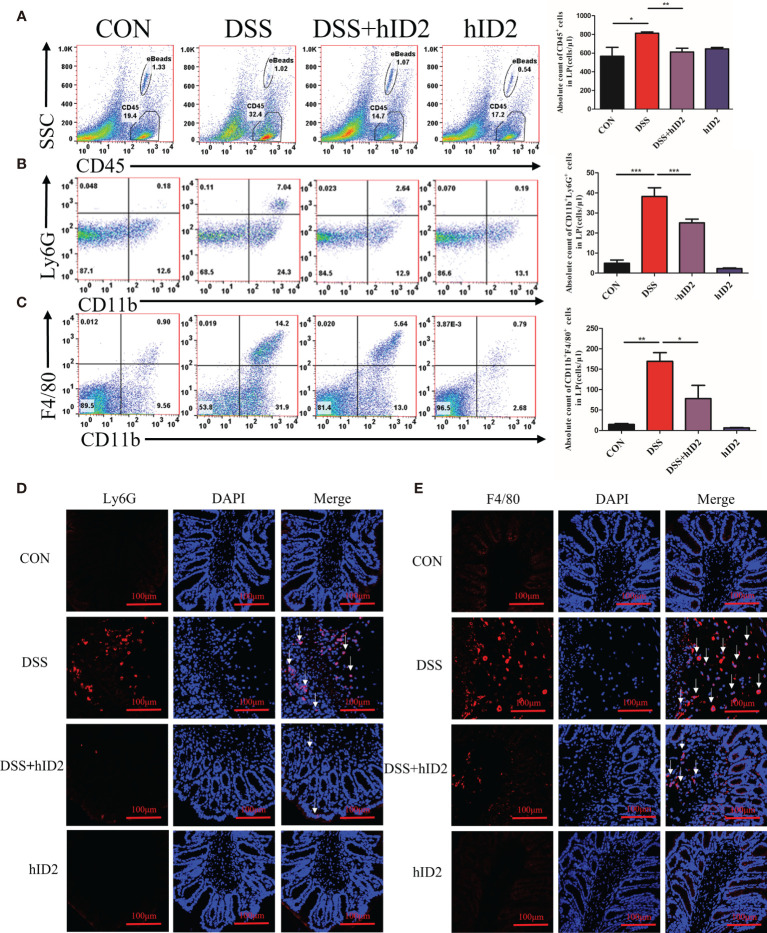
Recombinant human ID2 protein decreased inflammatory cell infiltration in the colonic lamina propria. **(A)** The absolute number of CD45^+^ cells in the colonic lamina propria (n=5). **(B)** The absolute number of neutrophils (CD11b^+^Ly6G^+^) gated from CD45^+^ cells in the colonic lamina propria (n=5). **(C)** The absolute number of macrophages (CD11b^+^F4/80^+^) gated from CD45^+^ cells in the colonic lamina propria (n=5). Sections of colonic tissues were immunostained with DAPI (blue) and **(D)** Ly6G antibodies (red) or **(E)** F4/80 antibodies (red) (400×) (n=3). The white arrows are for neutrophils or macrophages. The data are presented as the mean ± SEM. ^*^ *p* < 0.05, ^**^ *p* < 0.05, ^***^ *p* < 0.001.

Increased numbers of cells expressing CD45^+^ and CD11b^+^, which are reported surface markers of leukocytes, including neutrophils and macrophages, can be used to monitor inflammation (44–46). To further explore the protective effect of hID2, the absolute counts of CD45^+^ cells, neutrophils (CD11b^+^Ly6G^+^), and macrophages (CD11b^+^F4/80^+^) in the LP of the colonic mucosa were determined by flow cytometry. The results showed significant increases in CD45^+^ cells (814 ± 13, cells/μl), neutrophils (38 ± 4, cells/μl) and macrophages (169 ± 21, cells/μl) in the DSS group and decreases in the numbers of these cells in the DSS+hID2 group (CD45^+^ cells: 607 ± 51; neutrophils: 25 ± 2; macrophages: 78 ± 32, cells/μl) (**Figures 5A–C**). Immunofluorescence analysis of F4/80 and Ly6G, which are markers of macrophages and neutrophils, respectively, also indicated increased expression in the DSS group, and hID2 treatment apparently reduced the infiltration of macrophages and neutrophils in the colon (**Figures 5D, E**).

### Effect of Recombinant hID2 on Neutrophils and Macrophages in Peripheral Blood and the Balance of Treg/Th17 Cells in the Spleen

**Figure 6:**
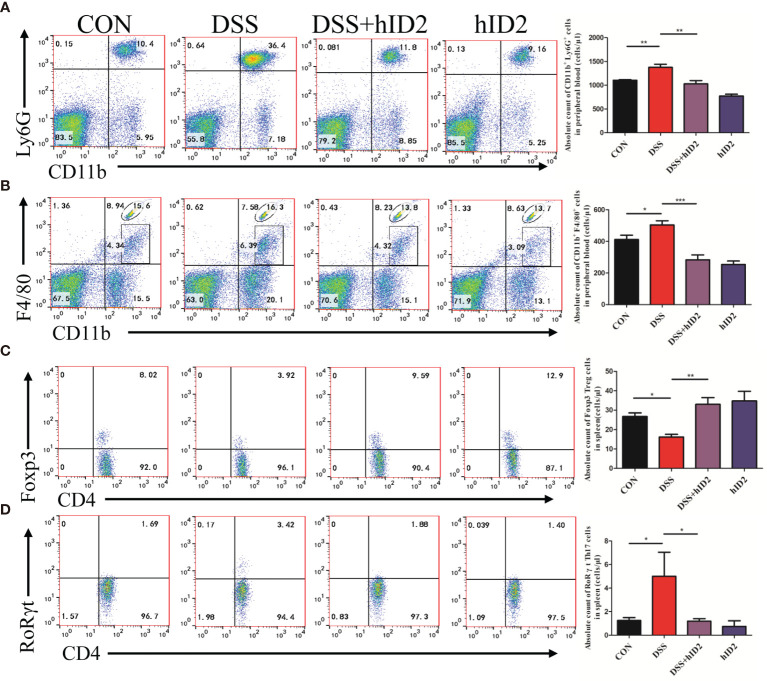
Recombinant human ID2 protein reduced the frequencies of neutrophils and macrophages in the peripheral blood of mice and maintained the balance of Treg/Th17 cells in the spleen. The absolute number of **(A)** neutrophils (CD11b^+^Ly6G^+^) and **(B)** macrophages (CD11b^+^F4/80^+^) gated from CD11b^+^ cells in the peripheral blood of mice (n=5). The absolute number of **(C)** Treg cells (CD4^+^Foxp3^+^) gated from CD4^+^ T cells and **(D)** Th17 cells (CD4^+^RoRγt^+^) gated from CD4^+^ T cells in the spleen (n=5). The data are presented as the mean ± SEM. ^*^ *p* <0 .05, ^**^ *p* < 0.05, ^***^ *p* < 0.001.

Furthermore, we also examined changes in cells in the peripheral blood of mice using flow cytometry. The absolute counts indicated that neutrophils and macrophages were decreased in the DSS+hID2 group (856 ± 128 and 168 ± 36 cells/μl, respectively) in comparison with the DSS group (1865 ± 282 and 472 ± 41 cells/μl, respectively) (**Figures 6A, B**), which was concomitant with an influx of CD11b^+^Ly6G^+^ neutrophils and CD11b^+^F4/80^+^ macrophages in the LP of the colonic mucosa (**Figure 5**). Treg cells are considered to be an important subset of CD4^+^ lymphocytes that play a protective role in DSS-induced colitis in mice. The balance of Treg/Th17 cells is vital for controlling colitis. To investigate whether hID2 treatment had an effect on the balance of Th17/Treg cells, the Treg/Th17 ratio in the spleen was examined. The results showed that the absolute number of Treg (CD4^+^Foxp3^+^) cells was lower in the DSS group (16 ± 1, cells/μl) than in the CON group (28 ± 1, cells/μl) (**Figure 6C**, *p*<0.001), while these cells were increased by hID2 treatment (36 ± 3, cells/μl). The absolute number of Th17 (CD4^+^RORγt^+^) cells was higher (**Figure 6D**, *p*<0.05) in the DSS group (5 ± 2, cells/μl) than in the CON group (1 ± 0, cells/μl) and DSS+hID2 group (1 ± 0, cells/μl). Interestingly, the numbers of Treg and Th17 cells were almost the same in the CON group and DSS+hID2 group. Thus, hID2 treatment significantly maintained the Th17/Treg cell balance in UC mice.

### The Protective Effect of hID2 on Colitis Was Not Dependent on the Gut Microbiota

**Figure 7:**
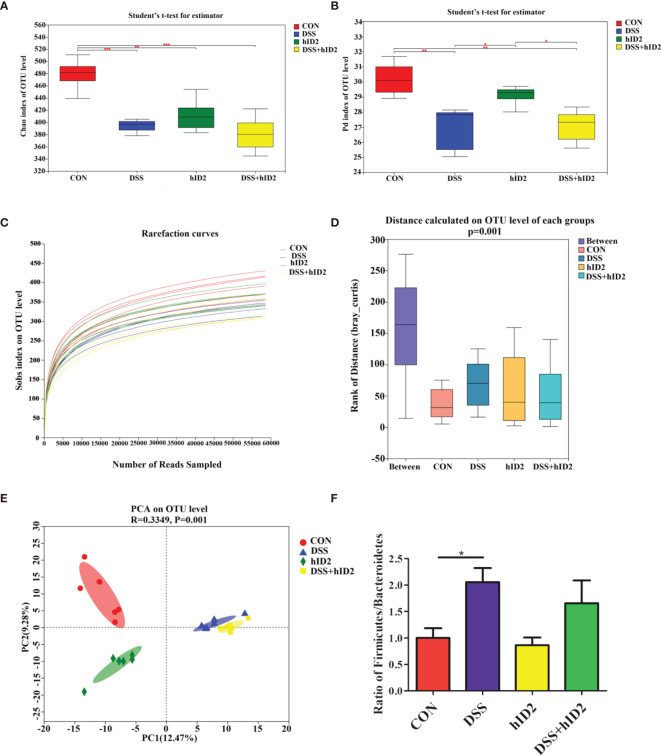
The protective effects of hID2 on colitis was not dependent on the gut microbiota. Fresh faeces were collected from mice on Day 8, and DNA was extracted for 16S rDNA sequencing. The alpha diversity index of the gut microbiota with the **(A)** Chao index and **(B)** phylogenetic diversity (Pd) index at the OTU level. **(C)** Rarefaction curves of the observed richness index (Sobs) at the OTU level. **(D)** The rank of distance on the OTU level of each group. **(E)** PCA of the intestinal microbiota at the OTU level in mice. PC1 and PC2 exhibited 12.47% and 9.28% of the variation, respectively. **(F)** The *Firmicutes/Bacteroidetes* (F/B) ratio at the phylum level. The ratio of F/B results is presented as the mean ± SEM. n=6 for each treatment. ^*^ *p* < 0.05, ^**^ *p* < 0.01, ^***^ *p* < 0.001.

To explore whether hID2 plays a protective role by regulating the intestinal microbiota, we analysed faecal samples using 16S rDNA high-throughput sequencing. As shown in **Figures 7A, B**, alpha diversity in the DSS group and hID2 group was obviously lower than that in the CON group, as indicated by the Chao index and phylogenetic diversity (Pd) index, and there was no significant difference between the DSS+hID2 group and the DSS group. The rarefaction curve of the observed richness (Sobs) index tended to be flat, indicating that the sequencing data were reasonable (**Figure 7C**). The analysis of similarities (ANOSIM) results showed that the differences between the groups were significantly greater than the differences within the groups, indicating that the experimental groupings were meaningful (**Figure 7D**). Moreover, at the OTU level, principal component analysis (PCA) revealed the overall structure of the intestinal microbiota in the different groups. The DSS group and hID2 group were obviously separated from the CON group; however, the DSS+hID2 group was still clustered together with the DSS group (**Figure 7E**). Relative abundance of bacteria at phylum levels (>1%) in the CON, DSS, DSS+hID2 and hID2 group mice was shown in **Table S2**. In addition, the *Firmicutes*/*Bacteroidetes* ratio (F/B) was higher in the DSS group than in the CON group (*p*<0.05), while the F/B ratio remained unchanged by hID2 treatment (**Figure 7F**). Therefore, although hID2 affected the diversity of the intestinal microbiota to some extent, it did not cause a change in the intestinal microbiota in DSS-induced murine colitis, suggesting that hID2 does not play a protective role by regulating the intestinal microbiota.

### Recombinant hID2 Was Internalized Mainly by Neutrophils and Inhibited Neutrophil Functions

**Figure 8:**
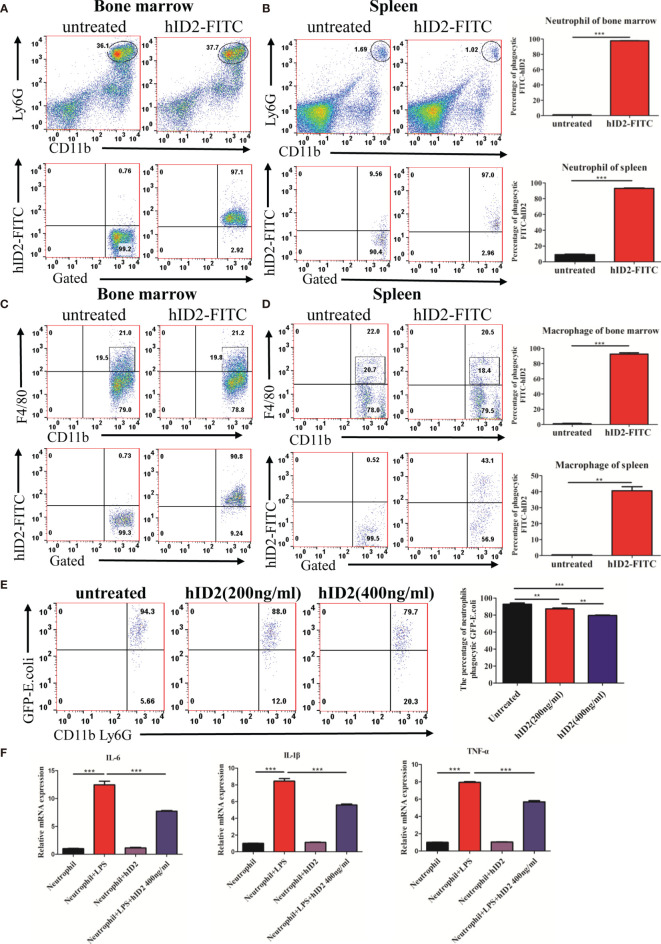
hID2 was efficiently endocytosed by neutrophils and macrophages and affected the function of neutrophils. The percentage of phagocytic neutrophils in the bone marrow **(A)** and spleen **(B)** that took up FITC-hID2. The percentage of phagocytic macrophages in the bone marrow **(C)** and spleen **(D)** that took up FITC-hID2. **(E)** Percentage of GFP-labelled *Escherichia coli* (*E. coli*) that were phagocytosed by neutrophils in the peripheral blood of mice in the presence of hID2. **(F)** The mRNA levels of IL-1β, IL-6 and TNF-α in LPS-stimulated neutrophils in the bone marrow in the presence or absence of hID2 treatment. n=3 for each treatment. The data are presented as the mean ± SEM. ^**^ *p* < 0.01, ^***^ *p* < 0.001.

To shed light on the mechanism by which hID2 protects against colitis, we first investigated the effect of hID2 on Caco-2 cells, which are human intestinal cell lines that have been widely used as a model of the intestinal barrier (47). We treated Caco-2 cells with different concentrations of DSS and found that the proliferation of Caco-2 cells was decreased with increasing DSS concentrations (**Figure S4A**). Although hID2 did not affect the proliferation of Caco-2 cells, it did not exert a protective effect against DSS-induced damage to Caco-2 cells *in vitro* (**Figures S4B, C**). Therefore, we explored the effect of hID2 on immune cells. The bone marrow and spleen were separated from mice and incubated with different concentrations of FITC-labelled hID2, and the results showed that FITC-labelled hID2 could be internalized efficiently by bone marrow and spleen cells, especially in the bone marrow (**Figures S5A, B**). Further studies showed that the hID2 protein was mainly internalized by neutrophils and macrophages, and the internalization rates were up to 95% in neutrophils collected from mouse bone marrow and spleen; however, the internalization rate in macrophages was slightly different, and the internalization rate in macrophages in bone marrow was also greater than 90% but only approximately 40% in macrophages from the spleen (**Figures 8A–D**). Therefore, we next focused on exploring the effects of hID2 on neutrophil function. We isolated neutrophils from the bone marrow of mice using the Percoll isolation method, and the purity was determined by flow cytometry. The purity of CD11b^+^Ly6G^+^ neutrophils was greater than 90% after isolation (**Figure S6A**). After coculture with *Escherichia coli* containing the GFP^+^ plasmid (**Figure S6B**), hID2 decreased the phagocytosis efficiency of neutrophils in a dose-dependent manner (**Figure 8E**). Additionally, hID2 treatment decreased the mRNA levels of IL-6, IL-1β and TNF-α in LPS-stimulated neutrophils (**Figure 8F**); thus, hID2 inhibited the function of neutrophils and exerted anti-inflammatory effects.

### The Protective Effect of hID2 Against Colitis Was Dependent on Neutrophils

**Figure 9:**
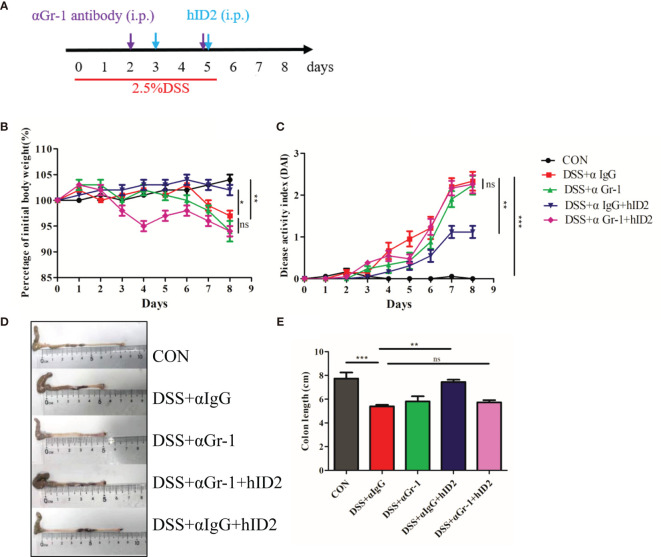
The protective effect of hID2 on DSS-induced colitis was dependent on neutrophils. **(A)** The experimental procedure; the purple arrow indicates *i.p.* injection of anti-Gr-1 or anti-IgG antibodies on day 2 and 5, and the blue arrow indicates *i.p.* injection of the hID2 protein on day 3 and 5. **(B)** Daily body weight changes. **(C)** Disease activity index (DAI) during the experiment. **(D)** Representative image of the colon and **(E)** colon length (ns, no significant difference). The data are presented as the mean ± SEM (n=8). ^*^ *p* < 0.05, ^**^ *p* < 0.01, ^***^ *p* < 0.001.

The protective effect of hID2 against colitis is mainly dependent on neutrophils. To further investigate the underlying mechanism of hID2, neutrophils were depleted in mice using anti-αGr-1 antibody (**Figures S7** and **9A**). Compared with mice in the DSS+αIgG group, mice in the DSS+αIgG+hID2 group had less weight loss, lower DAI sores, and longer colon lengths (**Figures 9B–E**), which was consistent with previous results (**Figure 3**). Interestingly, hID2 lost its protective effect against colitis in the absence of neutrophils, and there were no significant differences in body weight, DAI scores or colon lengths among the DSS+αIgG group, DSS+αGr-1 group and DSS+αGr-1+hID2 group (**Figures 9B–E**). These results suggested that the protective effects of hID2 against colitis was mainly dependent on neutrophils.

### hID2 Inhibited NF-κB Activation in LPS-Stimulated Neutrophils

**Figure 10:**
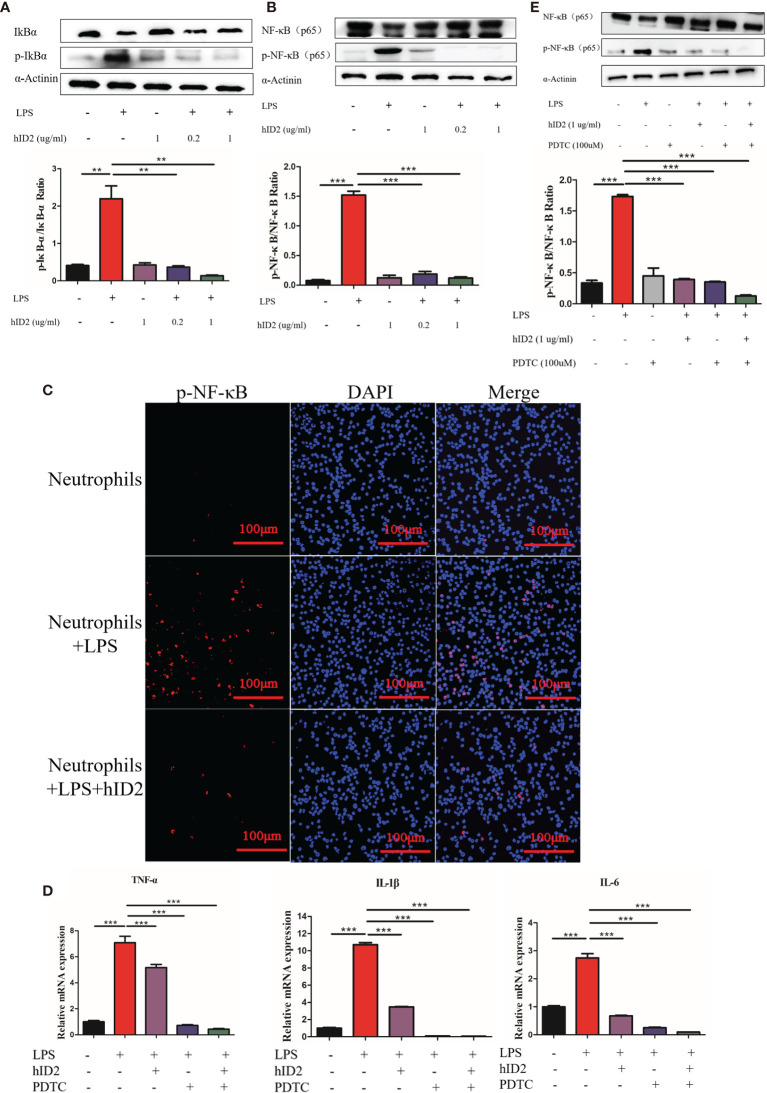
hID2 suppressed proinflammatory cytokines by inhibiting the activation of NF-κB pathway in LPS-stimulated neutrophils. **(A)** Representative immunoblot showing p-IκBα/IκBα in LPS-stimulated neutrophils with or without hID2 treatment. The relative protein levels of p-IκBα/IκBα are shown on the right. **(B)** Representative immunoblot showing p-NF-κB/NF-κB in LPS-stimulated neutrophils with or without hID2 treatment. Relative p-NF-κB/NF-κB protein levels are shown on the right. **(C)** Neutrophils from the bone marrow stimulated by LPS were immunostained with DAPI (blue) and p-NF-κB antibodies (red) (400×) (n=3). **(D)** The mRNA levels of IL-6, TNF-α and IL-1β in LPS-stimulated neutrophils in the bone marrow in the presence of hID2, PDTC or their combination (n=3). **(E)** Representative immunoblot showing p-NF-κB/NF-κB in LPS-stimulated neutrophils of bone marrow in the presence of hID2, PDTC or their combination. Relative p-NF-κB/NF-κB protein levels are shown on the right. The experiment was repeated three times independently. The data are presented as the mean ± SEM. ^**^ *p* < 0.01, ^***^ *p* < 0.001.

Modulation of the NF-κB signalling pathway in neutrophils is the main mechanism by which hID2 protects against colitis. In LPS-stimulated neutrophils, the protein level of total NF-κB was decreased, and the phosphorylation levels of IκB and NF-κB were increased in neutrophils, while hID2 treatment very effectively decreased the phosphorylation of IκB and NF-κB (**Figures 10A, B**), which was further confirmed by immunofluorescence analysis of p-NF-κB (**Figure 10C**). Contrary to expectations, it was found that the inhibition of NF-κB activation by hID2 could not be compromised by pyrrolidine dithiocarbamic acid (PDTC), a specific NF-κB inhibitor, and moreover, the inhibition of NF-κB activation even showed additive effects in the presence of PDTC and hID2. The phosphorylation of NF-κB and the production of inflammatory factors, such as IL-6, TNF-α and IL-1β, could be further inhibited by the combination of hID2 and PDTC (**Figures 10D, E**).

## Discussion

Ulcerative colitis (UC) is a major form of inflammatory bowel disease (IBD) that has been increasing in incidence in recent years (48). Due to the limitations and side effects of many current treatments, more effective agents are urgently needed. In our study, we found that the expression of ID2 was decreased in the inflamed colon in UC patients and mice with DSS-induced colitis. According to our long-term experience in the study of DSS induced colitis in mice, in the pathological development of DSS induced colitis, the phenotype appeared on the fourth day after drinking water containing DSS, thus we chose the third day as the intervention time point. Oral administration will lead to degradation of hID2 protein so that we used intraperitoneal injection for treatment. In addition, due to hID2 is a small molecule protein with fast metabolism, we injected it for the second time on the fifth day. We found that exogenous supplementation with hID2 could effectively alleviate DSS-induced colitis by inhibiting the IκB/NF-κB pathway in neutrophils.

Recent studies have shown that not only does the adaptive immune response play a role in the pathogenesis of IBD but the innate immune response is also equally important in inducing intestinal inflammation in patients (4). In IBD patients, the balance of regulatory T cells (Tregs) and T helper cells (Th17) cells is disrupted, with fewer Treg cells and more Th17 cells (49), which is consistent with our experimental results in DSS-induced colitis mice. Moreover, previous studies have shown that the recruitment and infiltration of neutrophils and macrophages, which are innate immune cells, promote inflammatory reactions by the secretion of proinflammatory cytokines and damage the integrity of the intestinal epithelial barrier (50). It was reported that the *Id2* gene could affect the differentiation of immune cells. Numbers of granulocyte/macrophage progenitors and matured neutrophils in the peripheral organs and bone marrow were higher in *Id2*-deficient mice (51), and mice lacking *Id2* and *Id3* could develop colitis (28, 29). In our study, ID2 was decreased in mice with DSS-induced colitis and UC patients; therefore, the decrease in ID2 expression might increase neutrophil and macrophage infiltration into colon tissue. Theoretically, exogenous supplementation with ID2 protein could reverse this phenomenon. In our study, we found that hID2 treatment slightly decreased the absolute counts of macrophages and neutrophils in peripheral blood compared to those of wild type mice and maintained the homeostasis of neutrophils and macrophages in the LP of DSS-induced colitis mice. In the DSS group, a significant increase in neutrophils and macrophages in the LP and peripheral blood of mice was found compared to CON group, indicating that the inflammatory status was increased in DSS-induced mice, and hID2 treatment alleviated DSS-induced colitis and decreased the numbers of infiltrating macrophages and neutrophils in the LP. Additionally, we found an imbalance in Treg/Th17 cells among spleen cells from DSS-induced mice, and exogenous supplementation with hID2 reversed the Treg/Th17 imbalance, suggesting a comprehensive improvement in colitis after hID2 treatment in DSS-induced mice.

The secretion of proinflammatory cytokines and impaired integrity of the colonic mucosal barrier are also reasons for IBD (52). Many proinflammatory cytokines, such as IL-6, IL-1β, TNFα, and IL-17, are increased in DSS-induced colitis and UC patients (53). *Muc2* is critical for maintaining the mucosal barrier as the first physical barrier, and the loss of the *Muc2* gene in mice results in the spontaneous development of colitis (54, 55). In addition, tight junction (TJ) proteins, which consist of claudin, occludin, junctional adhesion molecules and ZO family proteins, play important roles in preventing the immune response caused by pathogenic bacteria (56). In our study, we found that the levels of proinflammatory cytokines were increased in the DSS group but decreased significantly after treatment with hID2. Moreover, certain proinflammatory cytokines were less in the hID2 group compared to CON group. This may be due to two factors: on the one hand, *Id2* could affect the differentiation of immune cells (23–25), and on the other hand, hID2 could inhibit the activation of NF-κB pathway as shown in **Figure 10B**, thereby reducing the secretion of pro-inflammatory cytokines further. These protective effects were also reflected in the mRNA level of *Muc2* and the protein expression of claudin-1 and ZO-1. Salmonella invasion assays and FITC-dextran assays also confirmed that hID2 could reduce damage to the mucosal barrier. To explore the specific mechanism by which hID2 protects against mucosal damage, we treated Caco-2 cells with different concentrations of DSS *in vitro*. The results showed that Caco-2 cells were seriously damaged by increasing DSS concentrations, while hID2 did not play a protective role.

Intestinal microbiota dysbiosis is closely associated with UC. Thus, improving the intestinal microbiota has become a treatment strategy for intestinal inflammation. In this study, we found that the diversity index was decreased in DSS group mice compared with CON group mice; however, there were no differences between the DSS group and DSS+hID2 group. As expected, PCA showed that the composition of the DSS group and CON group was significantly different, while the DSS+hID2 group and DSS group were similar. These results suggested that the protective effect of hID2 on colitis was not dependent on the modulation of the microbiota.

To further explore the mechanism by which hID2 works, we focused on immune cells. First, we found that FITC-labelled hID2 could enter spleen cells and bone marrow cells in a concentration-dependent manner. As neutrophils and macrophages are the two major subsets with high phagocytic functions in bone marrow and spleen, we used FITC-labelled hID2 to investigate neutrophil and macrophage phagocytosis *in vitro*. The results showed that FITC-hID2 could be effectively phagocytosed by neutrophils and macrophages, especially by neutrophils. It was also reported that the ID2 protein could form a dimer (57), which was consistent with our expressed recombinant hID2, and the ID2 protein in cells could shuttle from the nucleus to the cytoplasm (58), but its detailed cytoplasmic function is not yet clear. Neutrophils are important for controlling colitis, and recent findings suggest that neutrophils are the major contributor to the development of UC (59); therefore, we sought to investigate the relationship between hID2 and neutrophils. Interestingly, the depletion of neutrophils with anti-Gr-1 antibodies completely abolished the protective effects of hID2 on DSS-induced colitis, suggesting that the hID2 protein exerts its protective effect by acting on neutrophils. However, the colon length was similar between DSS + anti-Gr-1 and DSS + anti-IgG groups, which indicated the damage caused by DSS might reach the limit of colon shorten. In the other hand, it was also indicated that the depletion of neutrophils with anti-Gr-1 antibody could not alleviate the DSS-induced colitis, suggesting the dual role of neutrophils in colitis. The depletion of neutrophils exacerbated DSS-induced colitis in our study, which was consistent with previous reports (60). The results indicated that alterations of neutrophil functions play an important role in the pathogenesis of colitis. Our further study showed that hID2 could significantly suppress the secretion of proinflammatory cytokines and the phosphorylation levels of IκB/NF-κB in LPS-stimulated neutrophils *in vitro*; moreover, the decrease in IκB/NF-κB phosphorylation could not be blocked by the inhibitor PDTC, and there was an additive effect on the downregulation of IκB/NF-κB phosphorylation when hID2 was combined with PDTC; thus, hID2 might be a new agent to enhance the inhibition of inflammation *via* the NF-κB signalling pathway. In our study, we also found that the ability of neutrophils to phagocytize GFP-labelled *E. coli* was partially inhibited by hID2 in a dose-dependent manner, which might be due to the inhibition of NF-κB activation, as the activation of NF-κB could lead to increased neutrophil phagocytosis (61).

In conclusion, our study explored a novel therapeutic strategy for treating UC by targeting innate immune cells with hID2. We found that the hID2 protein could effectively ameliorate DSS-induced acute colitis mainly by suppressing the activation of the NF-κB pathway in neutrophils. As the amino acid similarity between the human ID2 protein and mouse ID2 protein was approximately 99% (**Figure S1**), the anti-colitis effect of hID2 on mice considered to be effective in humans; thus, hID2 could be a promising agent for the treatment of UC. Our study also demonstrated the potential use of hID2 for its anti-inflammatory effects *via* the NF-κB pathway independent of PDTC-mediated inhibition. However, the deeper mechanism by which hID2 modulates the NF-κB pathway needs further investigation, and more animal models, such as DSS-induced chronic colitis and T cell transfer-induced colitis, are warranted to further assess the role of the hID2 protein in the treatment of UC.

## Data Availability Statement

The original contributions presented in the study are included in the article/**Supplementary Material**. Further inquiries can be directed to the corresponding authors.

## Ethics Statement

The animal study was reviewed and approved by the ethics committee of Xinxiang Medical University.

## Author Contributions

JR performed the experiments, analyzed and presented the data and wrote the manuscript. GZ and MW contributed to the study design, supervised the experiments and discussed results. DY, ML, WZ, and XX contributed to revision of the manuscript. YW, JZ, XJ, and PL participated in carrying out the experiment. MW and GZ conducted the experiments, data analysis, figure preparation and manuscript drafting. All authors contributed to the article and approved the submitted version.

## Funding

This work was supported by National Natural Science Foundation of China (No. 81872361, No. 31600109) and NSFC-Henan Union grant (No. U1904131), and the program for Science & Technology Innovation Talents in Higher Education of Henan Province (20HASTIT046).

## Conflict of Interest

The authors declare that the research was conducted in the absence of any commercial or financial relationships that could be construed as a potential conflict of interest.

## Publisher’s Note

All claims expressed in this article are solely those of the authors and do not necessarily represent those of their affiliated organizations, or those of the publisher, the editors and the reviewers. Any product that may be evaluated in this article, or claim that may be made by its manufacturer, is not guaranteed or endorsed by the publisher.

## Abbreviations

DSS, dextran sulfate sodium; UC, ulcerative colitis; IBD, inflammatory bowel diseases; ID2, Inhibitor of differentiation-2; hID2, recombinant human ID2 protein; TNF-α, tumour necrosis factor-α; IL-6, interleukin-6; IL-1β, interleukin-1β; IL-17, interleukin-17; IL-2, interleukin-2; IFN-γ, interferon gamma; GM-CSF, granulocyte-macrophage colony-stimulating factor; DAI, disease activity index; H&E, haematoxylin and eosin; LP, lamina propria; LPS, lipopolysaccharides; OTUs, operational taxonomic units; PCA, principal component analysis; NF-κB, nuclear factor kappa B; p-NF-κB, phosphorylated nuclear factor kappa B; PDTC, Pyrrolidine dithiocarbamic acid; IPTG, Isopropyl-β-D-thiogalactoside; *E. coli*, *Escherichia coli*.
