# Supplementary material for: Inhibitor of Differentiation-2 Protein Ameliorates DSS-Induced Ulcerative Colitis by Inhibiting NF-κB Activation in Neutrophils
Source: Front Immunol. 2021 Nov 4;12:760999. doi: 10.3389/fimmu.2021.760999 (PMC8599958; doi:10.3389/fimmu.2021.760999)
Supplement: Supplementary file 1 [file DataSheet_1.doc]

**Supplementary materials**

**Human *Id2* mRNA sequence (405 nt):**

ATGAAAGCCTTCAGTCCCGTGAGGTCCGTTAGGAAAAACAGCCTGTCGGACCACAGCCTGGGCATCTCCCGGAGCAAAACCCCTGTGGACGACCCGATGAGCCTGCTATACAACATGAACGACTGCTACTCCAAGCTCAAGGAGCTGGTGCCCAGCATCCCCCAGAACAAGAAGGTGAGCAAGATGGAAATCCTGCAGCACGTCATCGACTACATCTTGGACCTGCAGATCGCCCTGGACTCGCATCCCACTATTGTCAGCCTGCATCACCAGAGACCCGGGCAGAACCAGGCGTCCAGGACGCCGCTGACCACCCTCAACACGGATATCAGCATCCTGTCCTTGCAGGCTTCTGAATTCCCTTCTGAGTTAATGTCAAATGACAGCAAAGCACTGTGTGGCTGA

**Coded Amino Acid of human *Id2* gene：**

MKAFSPVRSVRKNSLSDHSLGISRSKTPVDDPMSLLYNMNDCYSKLKELVPSIPQNKKVSKMEILQHVIDYILDLQIALDSHPTIVSLHHQRPGQNQASRTPLTTLNTDISILSLQASEFPSELMSNDSKALCG

**BLAST detection of mouse *Id2* gene and human *Id2* gene：**


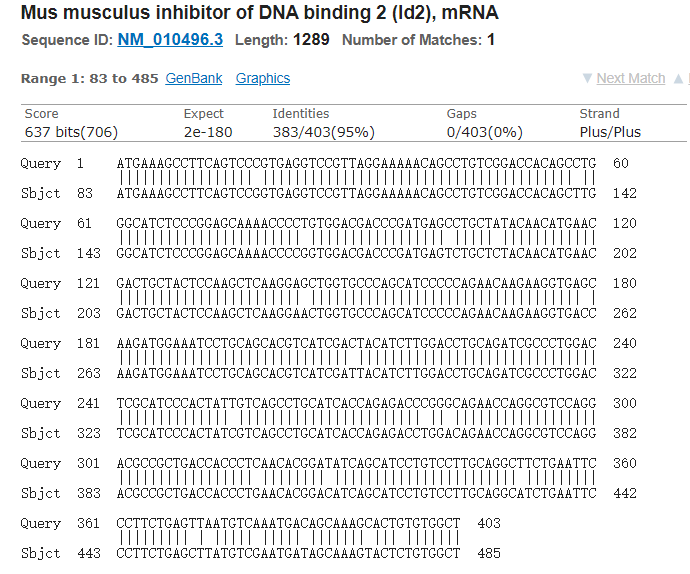


**Coded Amino Acid for BLAST between human *Id2* and mouse *Id2* gene, there is about 99% identities between the amino acid of human ID2 and mouse ID2 protein (132/134) as follows:**


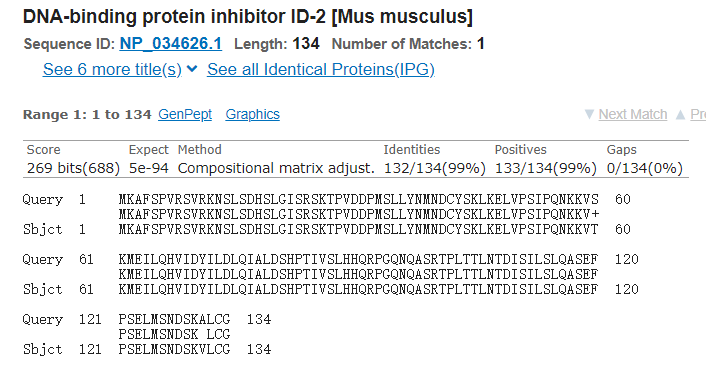


**Figure S1**. BLAST analysis of human Id2 and mouse Id2 gene and coded amino acid.


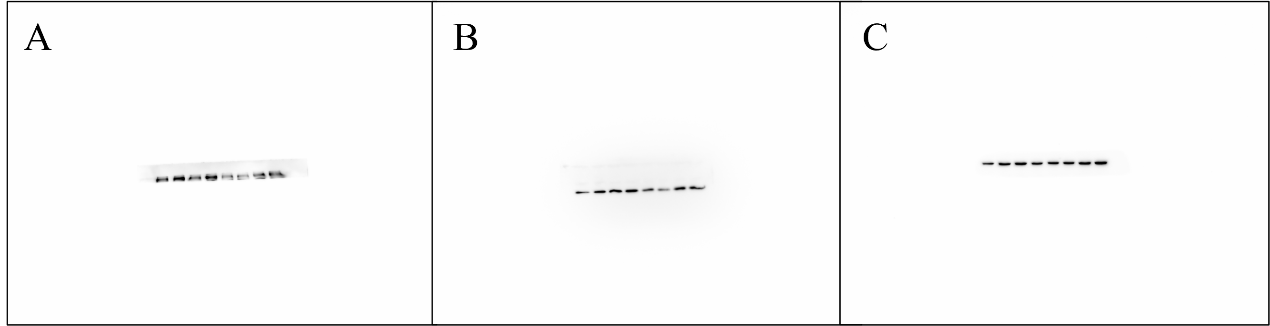


**Figure S2.** The uncropped and representative images of the original western blots. (A) The original western blot of ZO-1 (shown in manuscript Figure 4D). (B) The original western blot of Claudin-1 (shown in manuscript Figure 4D). (C) The original western blot of β-actin (shown in manuscript Figure 4D). n=5 for each treatment. The experiment was repeated three times independently.


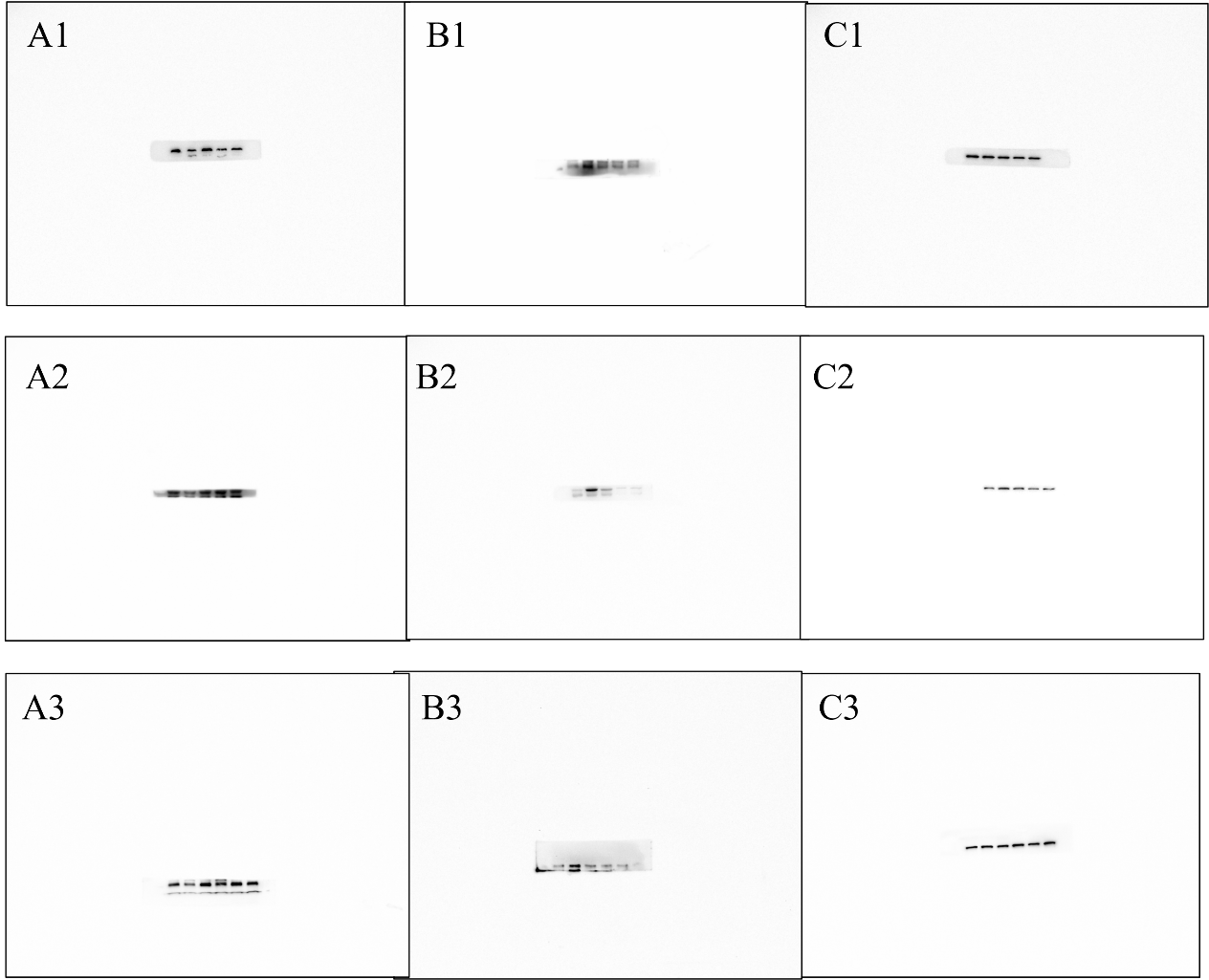


**Figure S3.** The uncropped and representative images of the original western blots. (A1) The original western blot of IκBα (shown in manuscript Figure 10A). (B1) The original western blot of p-IκBα (shown in manuscript Figure 10A). (C1) The original western blot of α-Actinin (shown in manuscript Figure 10A). (A2) The original western blot of NF-κB (p65) (shown in manuscript Figure 10B). (B2) The original western blot of p-NF-κB (p-p65) (shown in manuscript Figure 10B). (C2) The original western blot of α-Actinin (shown in manuscript Figure 10B). (A3) The original western blot of NF-κB (p65) (shown in manuscript Figure 10E). (B3) The original western blot of p-NF-κB (p-p65) (shown in manuscript Figure 10E). (C3) The original western blot of α-Actinin (shown in manuscript Figure 10E). The experiment was repeated three times independently.

**
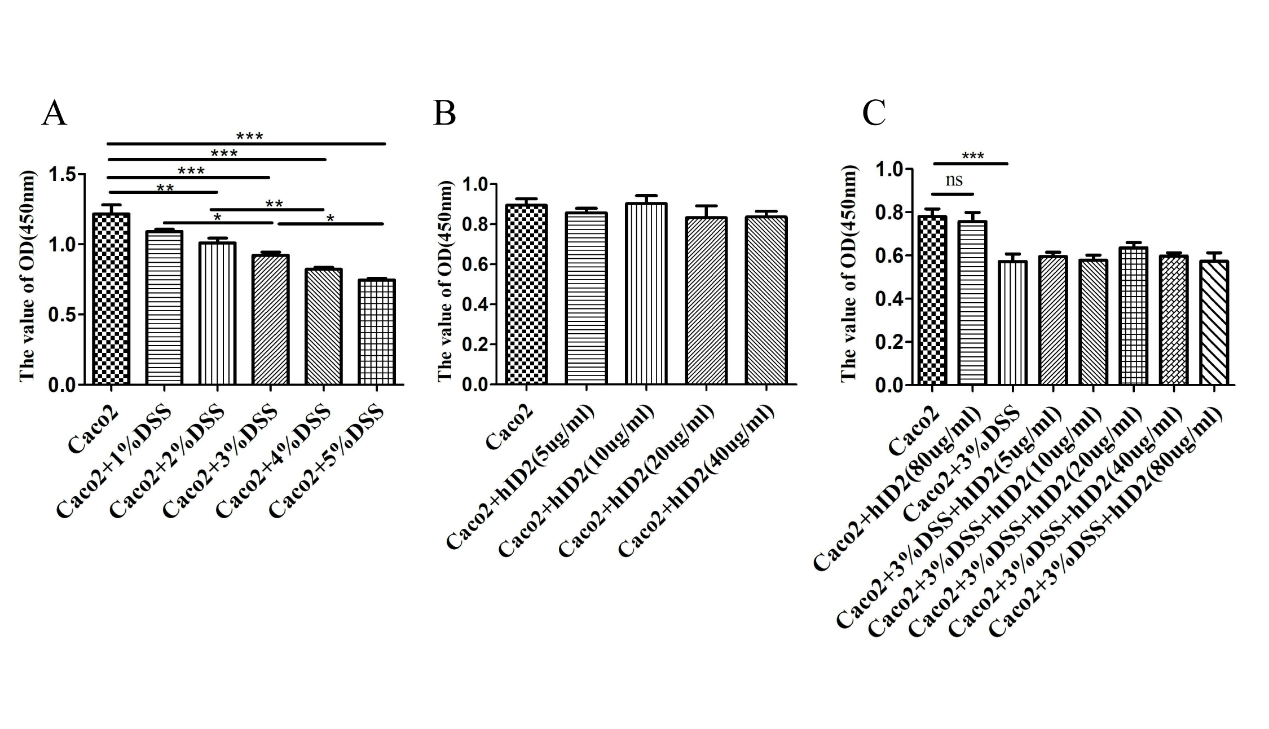
Figure S4**. hID2 could not reverse DSS-induced injury to Caco2 cells. (A) Effects of different concentrations of DSS on proliferation of Caco2 cells. (B) Effects of different concentrations of hID2 on proliferation of Caco2 cells. (C) The CCK-8 values of Caco2 cells treated with DSS and different concentrations hID2 alone or together, data indicated that hID2 could reverse the damaged caused by the DSS to Caco-2 cells *in vitro*. n=3 for each treatment. The experiment was repeated three times independently. Data are presented as mean±SEM. ^*^*p*<0.05, ^**^*p*<0.01, ^***^*p*<0.001.


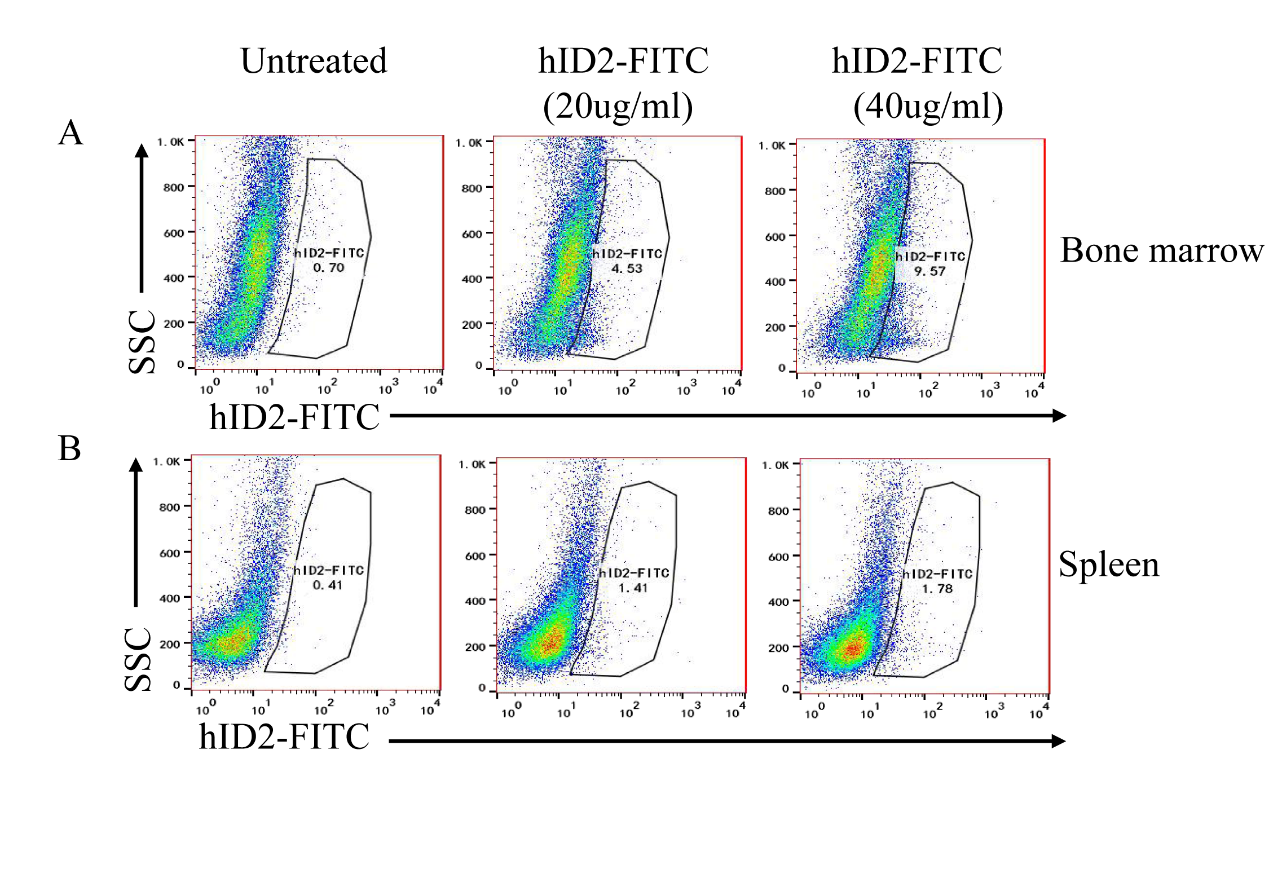


**Figure S5.** hID2 endocytosed by bone marrow and spleen in a dose dependent manner. (A) The percentage of FITC-hID2 in bone marrow cells. (B) The percentage of FITC-hID2 in spleen cells. n=3 for each treatment. The experiment was repeated three times independently.


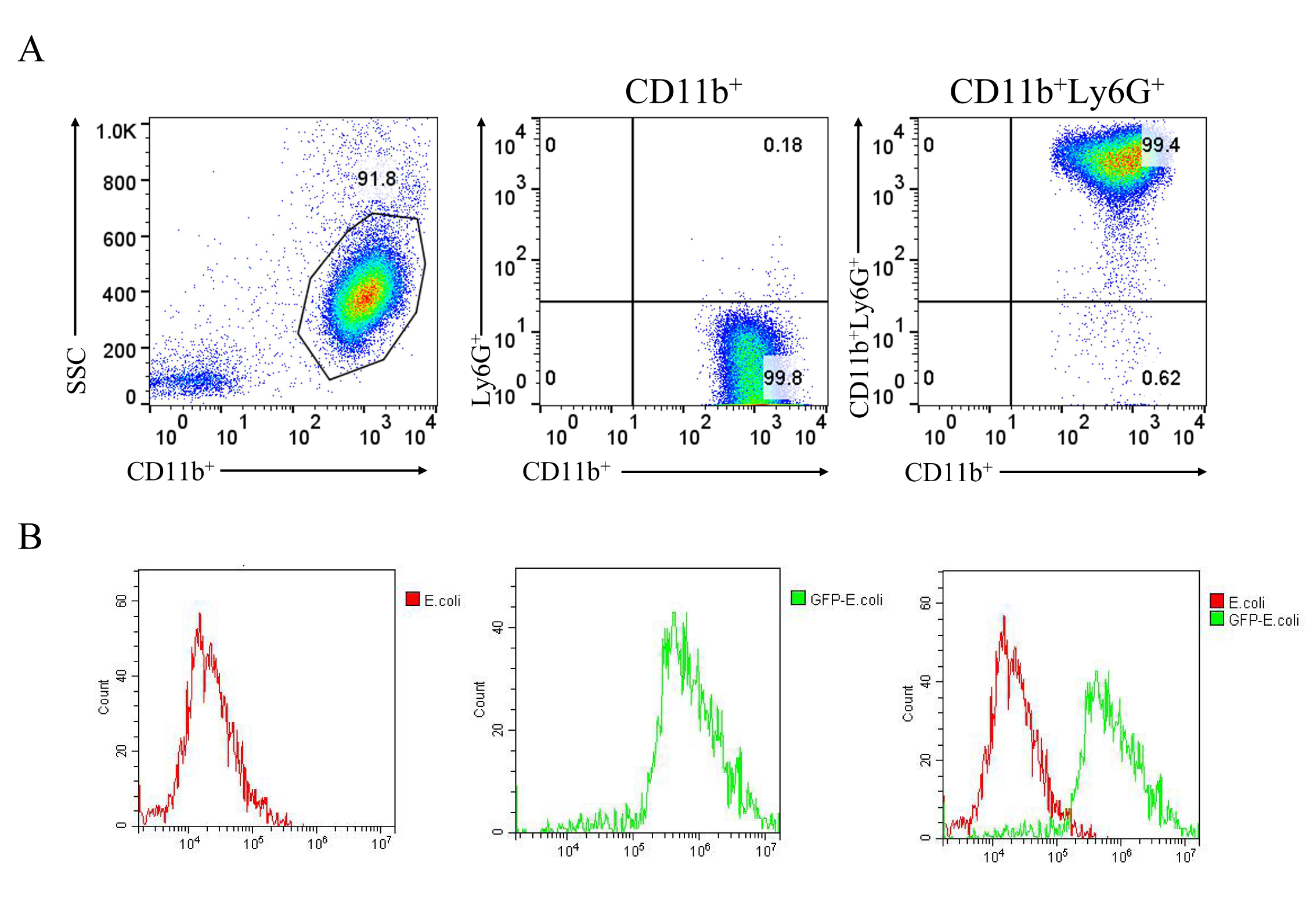


**Figure S6.** The purity of neutrophils isolated from bone marrow and identification of GFP labeled *Escherichia coli* (*E. coli*). (A) Flow cytometric profiles of neutrophils (CD11b^+^Ly6G^+^) isolated from bone marrow of mice. (B) Flow cytometric profiles of GFP labeled *E. coli*. n=3 for each treatment. The experiment was repeated three times independently.


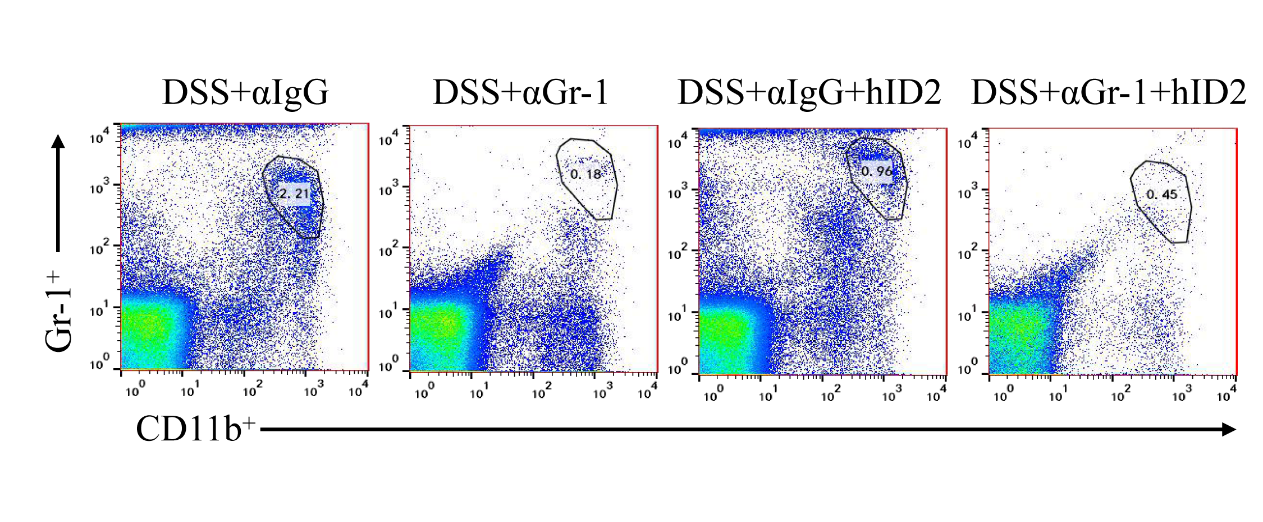


**Figure S7.** Neutrophils were effectively depleted by αGr-1 antibody in mice. Representative flow cytometric profiles of neutrophils (CD11b^+^Gr-1^+^) from peripheral blood of mice. Data indicated the anti-Gr-1 antibody could efficiently remove the neutrophils in the mice. n=3 for each treatment

**Table S1**. Primers used in the real-time PCR assays.

| Gene | Primer Sequences (5’-3’) |
| --- | --- |
| Muc-2  Forward  Reverse  TNF-α  Forward  Reverse  IL-1β  Forward  Reverse  IL-6  Forward  Reverse  IL-17  Forward  Reverse  IL-2  Forward  Reverse  IFN-γ  Forward  Reverse  Id-2  Forward  Reverse  GAPDH  Forward  Reverse | GCCTGGCTCTAATATGGA  TACTGCTTGTGAGGTGGG   CCCTCACACTCAGATCATCTTCT  GCTACGACGTGGGCTACAG  GCAACTGTTCCTGAACTCAACT  ATCTTTTGGGGTCCGTCAACT  TAGTCCTTCCTACCCCAATTTCC  TTGGTCCTTAGCCACTCCTTC  TGAGCAACTATTCCAAACCAGC  CGCAGCTCTAGGAGCATGTG  CCTGAGCAGGATGGAGAATTACA  TCCAGA ACATGCCGCAGAG  TCAAGTGGCATAGATGTGGAAGAA  TGGCTCTGCAGGATTTTCATG  ATGAAAGCCTTCAGTCCGGTG  AGCAGACTCATCGGGTCGT  TCTGGAAAGCTGTGGCGTGAT  GCCAGTGAGCTTCCCGTTCAG |

**Table S2.** Relative abundance（＞ 1%） of bacteria at phylum levels in the CON, DSS, DSS+hID2 and hID2 group mice.

| Phylum | CON | DSS | hID2 | DSS+hID2 |
| --- | --- | --- | --- | --- |
|  | Relative abundance(%) | | | |
| Firmicutes | 44.00±2.52 | 60.20±3.51^*^ | 36.75±2.78 | 53.40±4.87 |
| Bacteroidetes | 53.67±2.96 | 38.85±3.04^*^ | 58.75±4.25 | 48.13±3.49 |
| Proteobacteria | 0.61±0.07 | 0.83±0.35 | 0.56±0.25 | 1.01±0.30 |
| Deferribacterota | 0.38±0.13 | 0.77±0.29 | 0.11±0.07 | 0.65±0.11 |
| Actinobacteriota | 0.81±0.17 | 0.22±0.09 | 1.05±0.28 | 0.40±0.07 |
| Cyanobacteria | 0.50±0.16 | 0.06±0.01 | 1.52±0.79 | 0.01±0.00 |

n= 6 for each group. **p*<0.05 *vs.* the CON group.
